# Correspondence between Expected, Perceived, and Measured Effects of BoNT-A Treatment in Calf Muscles among Children and Adolescents with Cerebral Palsy: A Mixed Methods Study

**DOI:** 10.3390/jcm13051453

**Published:** 2024-03-02

**Authors:** Rannei Sæther, Ann-Kristin Gunnes Elvrum, Siri Merete Brændvik

**Affiliations:** 1 Regional Centre for Child and Youth Mental Health and Child Welfare, Department of Mental Health, Faculty of Medicine and Health Sciences, Norwegian University of Science and Technology, 7130 Trondheim, Norway; 2 Department of Rehabilitation Science and Health Technology, Faculty of Health Sciences, OsloMet—Oslo Metropolitan University, P.O. Box 4, St. Olavsplass, 0130 Oslo, Norway; 3 Department of Neuromedicine and Movement Science, Faculty of Medicine and Health Sciences, Norwegian University of Science and Technology, 7130 Trondheim, Norway; ann-kristin.elvrum@ntnu.no (A.-K.G.E.); siri.merete.brandvik@ntnu.no (S.M.B.); 4 Clinic of Rehabilitation, St. Olav’s Hospital, Trondheim University Hospital, 7130 Trondheim, Norway

**Keywords:** measurement, perception, expectation, energy expenditure, child-centered care, parallel database design

## Abstract

(1) **Background:** Our study explores the relationship between expected, perceived, and measured effects of botulinum toxin A (BoNT-A) treatment and saline (placebo) in children and adolescents with cerebral palsy (CP) in the calf muscles of 20 children and adolescents with cerebral palsy (CP), aged 4–15 years, using the Gross Motor Function Classification System (GMFCS) I–II. (2) **Methods:** A mixed methods parallel database design was used. Quantitative and qualitative data were collected at baseline and four weeks after treatment. The primary quantitative measure was *gross energy cost (EC)* during walking, obtained from a 5-Minute Walk Test (5MWT), while qualitative semi-structured interviews were performed with each parent and child/adolescent individually. (3) **Results:** Four weeks after treatment, we did not find any correspondence between expected, measured, and perceived effects. Interestingly, parental perceptions of treatment effects were more consistent than the measured outcomes. We also observed a connection between parental treatment expectations and perceived effects, often related to reduced energy expenditure. Children tended to view their parents as treatment experts and had fewer expectations and perceptions themselves. (4) **Conclusions:** These findings support the importance of child-centered care, which entails actively listening to children’s expectations and perceptions during the treatment process.

## 1. Introduction

Cerebral palsy (CP) is the most common cause of physical disability in childhood, resulting from damage to the developing brain. It has a reported point prevalence of 1.6 per 1000 livebirths in high income countries [1]. According to the definition provided by Rosenbaum et al. in 2007, *CP is a group of permanent disorders of the development of movement and posture*, *causing activity limitation*, *that are attributed to non-progressive disturbances that occurred in the developing fetal or infant brain. The motor disorders of CP are often accompanied by disturbances of sensation*, *perception*, *cognition*, *communication*, *and behaviour*, *by epilepsy*, *and by secondary musculoskeletal problems* [2]. The primary motor disorders associated with the condition include spasticity, muscle weakness, and reduced selective motor control. Additionally, individuals with CP frequently encounter secondary musculoskeletal issues, such as bony malformations and contractures [3]. While approximately 56% of children with CP can walk unaided [4], they still face varying degrees of limitations compared to their typically developing peers. These limitations may manifest as reduced walking speed [5], impaired balance [6], and increased energy costs during walking [7]. These deficits likely contribute to musculoskeletal pain and fatigue in children and adolescents with CP [8]. Probably as a result, individuals with CP are reported to be less physical active and participate less frequently in social activities than their peers [9,10].

Spasticity, being the most common neurological sign and present in as much as ~88% of cases [4], has been a main target in CP management of impairment level. This is based on the clinical assumption that spasticity hinders activity, although it is clinically accepted that spasticity also sometimes may be important for maintaining function (i.e., grip function). However, scientific evidence supporting an association between spasticity and activity levels is limited and inconsistent [11,12,13]. Intramuscular injections of botulinum toxin A (BoNT-A) were introduced in the 1990s and represented a breakthrough in spasticity treatment. The effectiveness of BoNT-A on reducing spasticity is well documented [14] and currently BoNT-A treatment is offered to as much as 55% of all children with spastic CP, with about half of these being independent walkers [15]. The calf muscles are the most treated muscles among these children [16], with the main goal being to correct spastic equinus gait [17]. Although the effects of these treatments on reducing spasticity are well documented, the evidence concerning the impact on walking and overall performance is still limited and inconclusive [18]. This discrepancy may be attributed, at least in part, to the choice of outcome measures [7]. Given the role of the ankle joint and the calf muscles in walking and energy transfer [19], the use of cardiorespiratory outcomes is relevant. Yet, only a few studies have employed cardiorespiratory measures that reflect walking ease, yielding conflicting results that are likely due to methodological differences [20,21,22]. 

When studying the effect of an intervention, it is important that contextual effects are taken into consideration [23]. Previous studies of the effect of BoNT-A treatment primarily consisted of research with quantitative approaches, measured effects, while only a few qualitative studies have explored caregivers’ experiences. Caregivers have reported positive effects, including reduced spasticity, improved motor function, increased happiness, and activity levels in their children [24]. It is important to note that caregivers also mentioned both benefits and drawbacks associated with the treatment [24,25,26]. Notably, previous studies have predominantly involved caregivers, but there is increasing interest in recent years to include children themselves in research. This shift acknowledges children’s rights (United Nations General Assembly) and recognizes their unique perspectives, experiences, and voices that should be considered [27].

Another aspect to consider is the impact of caregivers’ and children’s expectations on the perceived treatment outcomes. Recognizing the significance of understanding patient expectations in healthcare is becoming increasingly important. Patient expectations encompass their beliefs, hopes, and assumptions regarding the outcomes they anticipate from the treatment they receive [28]. These expectations can have a substantial influence on health outcomes [29]. 

Therefore, this study aimed to investigate the perspectives of both parents and children, including their expectations and perceptions of treatment effects, as well as to measure the effects of the treatment. The specific focus was on exploring the correspondence between expected, perceived, and measured effects of BoNT-A in calf muscles among children and adolescents with CP. A mixed methods design was employed to gather comprehensive data. The findings of this research are relevant to professionals involved in the care and support of families with children and adolescents with CP. By gaining a deeper understanding of the correspondence between expectations, perceived effects and measured outcomes, healthcare providers can enhance their ability to tailor treatments and interventions to meet the specific needs of children and adolescents with cerebral palsy and their families.

## 2. Materials and Methods

Data presented are from a sub-study in the multicenter study Walking Easier with Cerebral Palsy (WE study) [30]. The WE study is an industry-independent, double-blinded, placebo-controlled superiority trial aiming to identify whether injections with BoNT-A in the calf muscles make walking easier, judged by energy cost (EC) during comfortable walking. Thus, the goal for the treatment was to identify if BoNT-A treatment in the calf muscles make walking easier. The WE study was conducted in a clinical setting involving all four health regions in Norway. Children with spastic CP, for whom the responsible physician had identified an indication for BoNT-A injections in the calf muscles, were invited to participate. Those who were enrolled were randomized to receive either injections with BoNT-A or 0.9% saline in the calf muscles. The allocation ratio was 1:1 and stratification was performed according to age and study center. The main inclusion criteria were (1) age 4–17.5 years, (2) Gross Motor Function Classification System levels I and II, (3) no BoNT-A injections in the lower limbs during the past 6 months, and (4) no orthopedic surgery to the lower limbs during the past 2 years. Assessments were made at four timepoints; baseline, 4 (P1), 12 (P2), and 24 (P3) weeks post injection with the primary endpoint at P2. The participants had regular follow-ups during the study period. In Norway, many children with CP receive physical therapy (PT) on a regular basis. Among children classified at GMFCS levels I–II, the frequency commonly varies from one to two times per week to less than once per month depending on the needs of the child [31]. This paper is based on data obtained from a subsample of the Norwegian participants at baseline and P1. For a more comprehensive description of the methods, see Brændvik et al. [30].

### 2.1. Design

**Figure 1:**
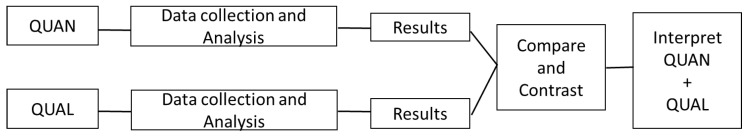
Parallel Database Design.

In this study, a mixed methods design with a convergent parallel approach and a parallel database design [31] was used. The parallel database design is structured so that the quantitative (QUAN) and qualitative (QUAL) data are collected separately but at the same time (concurrently). The data are also analyzed concurrently. The results are then converged by comparing and contrasting the data [32], see Figure 1.

A mixed methods design may give a better understanding of the experimental results by incorporating the perspectives of the individuals. By using this framework, we can gain deeper insights into the treatment effects of BoNT-A in children and adolescents with CP, generating knowledge that may provide useful information for research and clinical practice.

### 2.2. Participants and Recruitment

The participants of this sub-study, the chil and one of their parents, were strategically selected to ensure rich and varied insights into the research question. The selection criteria were the age of the children, both younger and older children, both mothers and fathers of these younger and older children, and various GMFCS levels. By including the children, we could obtain their first-person perspective. In addition, it was important to include the parents, as they play a significant role in the decision making concerning the child’s clinical follow-up, in the Norwegian Cerbral Palsy Follow-Up Program (CPOP). 

### 2.3. Ethical Considerations

The WE-study was approved by the Regional Ethical Committee (REC) for Medical Research in North Norway (2013/1195), ClinicalTrials.gov ID NCT2546999. We chose to perform individual interviews with parents and children separately to allow them to speak freely. To ensure that the children felt secure, the parents were seated in an adjacent room during the interviews, and the children had the option to enter the room and join their parents at any time.

### 2.4. Data Collection

#### 2.4.1. Qualitative Data—Interviews

The face-to-face semi-structured interviews were conducted by the first author (RS), on the same day as the baseline and P1 assessments. Two of the interviews at P1 were telephone interviews. All interviews were audio-recorded. The child and the parent (mother or father) were interviewed separately with open-ended questions. Children younger than six years were not interviewed, as they may have difficulty answering the questions in a meaningful manner. Neither the interviewer, parents, nor the children knew whether the child had received BoNT-A or saline. The interviews started with a general question: Can you tell me about what activities you/your child like to do? The interview guide continued with the following themes: (i) Motivation for/expectation to treatment: What are your expectations/wishes for how the treatment should work? (ii) Perceptions of treatment: Do you think you/your child received BoNT-A? Why, why not? How do you feel/observe/notice that the treatment works? How does the treatment affect/influence functions/activities you/your child can do/participate in? The participants were encouraged to discuss any additional relevant issues arising during the interview. 

#### 2.4.2. Quantitative Data—Measured Outcomes

The primary outcome measure was *gross energy cost* (*EC*) during walking, obtained from a 5-Minute Walk Test (5MWT) performed on a 45-m pathway at a self-chosen comfortable speed [33]. Simultaneous gas-exchange measurements, oxygen uptake (VO2) and carbon dioxide production (VCO2), were performed using a Metamax version II or IIIb (Cortex Biophysik GmbH, Leipzig, Germany) carried on the back with a facemask placed over the mouth and nose. The following formula was used to estimate EC:

$$Energy\;cost\left(J/kg/m\right)=\frac{\left(4.960\times RER+16.040\right)\times {VO}_{2}\;(mL/kg/min)}{walking\;velocity\;(m/min)}$$

with VO2 and VCO2 being the average oxygen uptake and carbon dioxide production, respectively, over a 1-min steady state period during the last two minutes of the walk test, relative to body weight (mL/kg/min). It has been reported that children with CP have EC during walking corresponding to 6.84 J/kg/m (SD: 2.0 J/kg/m) and that the minimally clinically important difference (MCID) of this measure is 0.464 J/kg/m (or 6.8%) [33]. 

The secondary outcome measures were: (i) *walking capacity* (*WC*), reflected as the distance walked during a 1-Minute Walk Test (1MWT) with the participant walking as fast as possible without running. The 1MWT is reported to be a valid measure of capacity in children with CP. For children at GMFCS levels I–II, the MCID is large if ≥9.0/8.3 m and medium if ≥5.6/5.1 m, respectively [34]; (ii) *calf pain intensity* (*PI*) *during the last two weeks*, *reported by proxy.* The parents were asked to respond to one question (“how much pain”) from the Child Health Questionnaire, on a six-point ordinal scale; no pain, very weak, weak, moderate, strong, and very strong [35]; and (iii) *perceived change in performance and satisfaction*, measured by the Canadian Occupational Performance Measure (COPM). The COPM is a reliable and valid semi-structured interview used to detect changes in self-perceived activity performance in the areas of self-care, productivity (i.e., school), and leisure [36]. Self-perceived performance and satisfaction of each activity problem were scored on a ten-point ordinal scale (1–10), where higher scores reflect greater performance and satisfaction, respectively. Two points or more are deemed MCID [37]. For a more comprehensive description of the methods, see Brændvik et al. [30].

### 2.5. Data Analysis

The sample size was determined by the qualitative analysis, in which recruitment ended once no further themes were emerging from the analysis. According to the parallel database design, see Figure 1, both the qualitative and quantitative data were analyzed before unblinding of the group belonging either to the Placebo group (P) or to the BoNT-A group (B). After separate analyses, the results were compared and contrasted.

#### 2.5.1. Qualitative Analysis

The interviews were transcribed verbatim and analyzed according to a systematic text condensation approach [38]. Qualitative Solutions for Research (QSR) International’s NVivo 10 software was used as the sorting tool (QSR International Pty Ltd., Melbourne, Australia, 2012). This method constitutes a pragmatic approach, despite being inspired by phenomenological ideas, and consists of four steps: (i) Total impression—preliminary themes; (ii) Identifying and sorting meaning units—from themes to codes; (iii) Condensation—from code to meaning; and (iv) Synthesizing—from condensation to descriptions and concepts [38].

Initially, the first author (RS) and a medical doctor (IT) searched for themes in the data, allowing issues that appeared to be significant for the parents and children to come to light. The next steps involved all of the authors and entailed a detailed coding characterized by the collapse of codes and subthemes and the subsequent condensation of meaning within each theme, resulting in the themes presented in the Results section. The initial coding was primarily developed inductively, based on the content of the data, while we found it appropriate to apply a phenomenological lens in our analytical interpretations of the empirical findings. This perspective emphasizes the study of subjective experiences and meanings as they are experienced by individuals [39]. This may be useful for exploring multifaceted experiences that are difficult to quantify or measure. 

Phenomenology, developed as a counter to the positivist emphasis on objectivity and measurability, was pioneered by Edmund Husserl, a German philosopher. This philosophical approach was further developed by Maurice Merleau-Ponty, a French philosopher who focused on embodied phenomenology [39]. In this context, the body is central in how we experience and generate knowledge about ourselves, others, things, and all the situations and activities we are involved in [40]. Therefore, our sensory impressions and expressions can be viewed as a dynamic and embodied interaction with the world [39]. Phenomenology seeks to understand an individual’s unique perspective of the world by exploring how they appear to the subject [39].

Before entering this project, all authors and collaborators had several years of clinical experience as physiotherapists, occupational therapist, and medical doctor, working with children and their families, as well as research experience in the field of descriptive, exploratory, and experimental study designs.

#### 2.5.2. Quantitative Analysis 

The data were analyzed using descriptive statistics (Statistical Package for Social Sciences software version 39). Changes from baseline to P1 for each participant were calculated (P1-Baseline). For EC, negative change score represents improved EC during walking. For WC, positive values represent improvement in terms of longer distance walked. For pain, negative values represent less pain. 

## 3. Results

In this section, we will begin by presenting the participants’ characteristics. Next, we will provide the analysis and interpretation of the qualitative data, followed by the analysis of the quantitative data. Finally, we will compare and contrast the findings.

### 3.1. Participants Characteristics 

A total of 20 parents and their children/adolescents in the WE-study [31] were recruited (from September 2015 to September 2017) for the qualitative interviews. No participants declined to take part in the study. At this stage, it was deemed that saturation had been achieved in the qualitative analysis. We considered including a larger number of children with GMFCS II in our study. However, we did not observe any distinct perspectives that differentiated them from those with GMFCS I. Ten females and 10 males aged 4–15 years, 12 mothers, and 8 fathers participated. Most of the children were classified as GMFCS level I and with unilateral (UL) CP, see Table 1. 

*The qualitative data*, presented in Table 2. comprised interviews with 20 parents and 18 children (two children were under the age of 6 and were not interviewed) at baseline. At P1, there were some missing data, resulting in qualitative data from 18 parents and 15 children. The reasons for missing data were either protocol deviation during treatment or poor quality of the audio recording, which we considered to be missing at random. After unblinding of group adherence, 11 parents and 10 children were in group P and 7 parents and 5 children were in group B, see Table 3.

*The quantitative data* comprised measured outcomes obtained from 20 children at baseline and 19 (one was excluded due to protocol deviation during treatment visit) children at P1. After unblinding of group adherence, 11 children were in group P and 8 children were in group B, see Table 3.

### 3.2. Qualitative Results

During our systematic text condensation [38], two main themes, *Expectations to treatment* and *Perceived effect*, became visible, and two categories emerged for the parents and the children in each theme (Table 2). In our description of the themes, we first present the voices of the parents and the children in each category, and then we present an interpretation of the theme’s knowledge contribution. The term botox is used in this section, as parents and children did not use the term BoNT-A.

#### 3.2.1. Expectations to Treatment 

The expectations to treatment differed somewhat between parents and children. Parents had expectations for changes in their child’s body to *approach normality*. They believed that achieving normality would help *prevent secondary problems*, *to improve functions*, *and enhance activities and participation*. Another important expectation was that the treatment would assist the child in *saving energy*. In contrast, the children mainly referred to the treatment as something they *were just doing.* However, they also expressed some expected *pros and cons* associated with the treatment.

##### Theme 1a: Parents’ Expectations

- Category 1: Approaching normality

*Preventing secondary problems* was important to the parents. They looked ahead and viewed botox as a treatment to prevent the development of contractures and deformities and believed that future problems could be avoided, with one parent stating: “If he uses his body in a more appropriate or normal way, he may avoid joint wear”. (parent 02). Pain was also a concern, and the parents hoped that botox treatment could prevent and alleviate it. One father expressed: “She probably has pain all the time, and when she says her foot hurts, it really hurts. The hope is that she becomes more relaxed, so she doesn’t feel it every day… That’s what I hope will happen”. (parent 4) Preventing future problems was also associated with aspects of being different or standing out. Many parents hoped that if their child’s body could develop more normally after the treatment, it would be easier for the child to fit in, thus reducing the risk of bullying and drop out from sports. One father articulated it as follows:

*“Perhaps she’ll avoid being bullied. It’s easy to target those who are perceived to have obvious weaknesses or visible functional impairment. Her mother is always afraid of this. We also hope she can keep up in sports for longer. It’s not enjoyable when everyone is much better than her at the ages of 12, 13, and 14, but this may not happen”.* (parent 9)

*Improvement in functions*, *activity*, *and participation*, like in sports, were also expectations that the parents emphasized. They believed that achieving normality, using the body in the “normal” way, would allow their child to maximize their potential. One parent explained the motivation for trying botox treatment: “We thought that if there was any possibility to get her from 90–100% in terms of speed, balance, and overall mobility, we wanted to try it”. (parent 9) Another parent expressed the hope for a transformative change: “She is starting to become a teenager, so I really hope they get her legs on the right track”. (parent 5) The parents associated normal development with improved performance in various activities, such as running faster, walking better and without splints, wearing nice shoes, jumping correctly, and competing at a high level. While improvement in activities was highlighted, participating in activities alongside others was also important. One mother explained the significance of participation:

*“The physiotherapist is very concerned that he must learn to ride a bike for the social aspect, so he can be with friends. Therefore, the focus is on enabling him to participate and be part of the group, to be one of the others”.* (parent 12)

- Category 2: Saving energy

*Saving energy* was the most prominent expectation of the botox treatment among the parents, which they believed would promote participation. To illustrate the duration of their concern, one mother remarked: “For the past year, we have been searching for the energy thief. We have undergone numerous medical checkups because she has such little energy”. (parent 13) Several parents viewed the reduction in spasticity as a means to make walking easier and more energy efficient. One father described it as follows: “Just before he puts his foot down, there is some triggering in the brain… The treatment will help him relax more when he walks, and he can save some energy”. (parent 16) The parents emphasized that saving energy and reducing fatigue could have a positive influence on the child’s well-being. One mother stated: “Maybe the quality of life can improve when you don’t get so tired; you’ll feel better”. (parent 13) Several parents described how the child’s low energy levels had an impact on everyday life. One mother said:

*“It’s typical that she can fall asleep when she gets home from school. And that she has to opt out in a way… She has good friends in the neighborhood, whom she has to exclude herself from for periods of time”.* (parent 13)

##### Theme 1b: Childrens’ Expectations

- Category 1: Just doing it

The children primarily trusted their parents’ observations and judgment when it came to botox treatment. They simply went along with it and were not fully aware of changes the parents noted, as they did not pay much attention to their bodies or to walking. One child expressed this sentiment by saying: “They say it’s going well, so I want to continue with the treatment. They think they see it, so I think it’s okay. I don’t ask how it looks”. (child 2) Another child remarked: “I don’t understand, but she must be seeing something I don’t know. I don’t walk and look at my feet all the time”. (child 18) Moreover, several children perceived botox treatment as something they were doing together with their parents. One of them said: “We only take it during winter because I tend to be less active then”. (child 18)

- Category 2: Pros and cons

Although the children generally let their parents make decisions, they expressed both expected pros and cons regarding the treatment. The pros were related to improvements in activities, particularly walking faster, longer, steadier, and wearing nice shoes. Several children also expected to experience less pain and reduced fatigue. These improvements were associated with anticipated changes in their bodies. One child described: “I think the muscle will work more on its own so that I don’t have to correct it all the time”. (child 5) Many children also hoped that various physical activities would become easier. One child expressed: “Maybe I’ll be better in football, and maybe I’ll run faster. Maybe ‘Tarzan catch and run’ will be more fun, I don’t know. I’m not an expert on the body. I actually don’t know”. (child 10) This child voiced some uncertainty about his understanding of his body, while other children were fully aware of the advantages their bodies could provide. They considered losing these advantages as a con of the treatment. This was illustrated by these quotes: “It’s a very good advantage that both sides are not equally strong because then there is something I can use the weaker side for…” (child 14) Another child stated: “The fact that I don’t step on my heel makes it easier for me to be a ballerina!” (child 6).

##### Interpretation of Parent’s and Children’s Expectations 

The parents’ approach of altering the child’s body to prevent future issues, by e.g., “getting the legs on the right track,” can be interpreted from a phenomenological perspective as treating the body as an object to be normalized or repaired. Phenomenology, on the other hand, understands the body as a whole, not just as separate parts, and emphasizes its integral role in our interaction with the environment [39]. Nevertheless, the parents also demonstrated a holistic view by recognizing the connection between the body and the child’s social environment. They emphasized that the treatment could improve the child’s ability to fit in and be a part of the group, particularly by reducing the energy expenditure through a more normal movement pattern. Their hope was that this would result in increased participation.

The children also expressed hopes that the treatment would bring improvements in their ability to participate in activities. However, they appeared to have a more passive role in the treatment process, simply going along with it without actively engaging or questioning it. This suggests that they viewed their parents as the experts, who had knowledge of their bodies. The children also emphasized that they were not overly concerned with how their feet looked, indicating that the appearance of their bodies was not a central focus for them. This aligns with Merleau-Ponty’s philosophy, which introduces the concept of the unthematized body—the implicit and non-objectified bodily dimension [40]. In our everyday activities like walking, running, or biking, we typically do not consciously contemplate the specific details of the movements involved. As embodied beings, we are consistently engaged in projects and bodily actions where the body recedes in the background, without having our attention [41].

The treatment process, with its focus on altering the child’s body, may contribute to thematizing the body, perceiving it as an object in need of change. However, it is important to note that the children themselves expressed positivity towards how their bodies currently functioned. They expressed concerns that the treatment might hinder their ability, for instance to be a ballerina, dancing on the tiptoes. The impression of the children’s passive role in the treatment process, coupled with the handling of their concerns, raises questions about the extent to which child-centered care is being practiced in this context. Child-centered care prioritizes the child’s perspectives, preferences, and well-being, ensuring their active involvement in decision-making processes that affect their own bodies and lives. This approach aligns well with a phenomenological perspective. However, Wågby et al., 2022 [42] pointed out an important distinction between a child’s perspective (as understood by adults) and the children’s perspective (i.e., the perspective of the children themselves) when “listening to children”.

#### 3.2.2. Perceived Effect of Treatment 

In this study, both parents and children provided their perspectives on the perceived effects of the treatment. The children’s viewpoints were captured from the first-person perspective, reflecting their own experiences and perceptions of the treatment. On the other hand, the parents’ perspective was obtained as observers, offering their observations and interpretations of the effects of the treatment on their children.

The following text describes how the parents perceived less stiffness in the muscles and changes that led to less stumbling. They also had a perception that the child had more energy and described how this influenced the ability for participation for the whole family. The children often referred to their parents, what they said, as they often did not perceive any change. They did, however, express that they could do more after the treatment.

##### Theme 2a: Parents’ Perceptions

- Category 1: Less stiff and stumbling

Many parents perceived changes following the treatment, some of which were visible while others were more subtle. One parent expressed: “I think he moves better… without being able to pinpoint exactly what it is” (parent 2). Another parent observed a noticeable change and said: “She hasn’t stumbled at all now, she’s better!” (parent 7). Over time, some parents also noticed changes in the calf muscles. One parent described: “In the second week, I noticed that his muscles were softer in a way. There was a different texture in the calf; it was quite relaxed in the middle” (parent 1). Several parents perceived that stiffness in the muscles had an impact on everyday life in various ways. One parent explained: “Before, she was stiffer, and it was more difficult for her to get started in the morning; she didn’t function properly! It’s easier for her to do things now” (parent 3).

- Category 2: More energy

As movements became easier, the parents perceived that their child had more energy to engage in activities and participate more actively. They attributed the increased activity level to more efficient use of energy. One parent expressed: “I think she can do the same activities more easily. She’s not moving faster, but there is less strain in what she does” (parent 7). Another parent shared a similar perception: “He’s more mobile when playing football, and I believe he gets less tired. It’s a more efficient use of energy” (parent 2). Some parents also noted that reduced pain after the treatment could contribute to the increased energy level. One parent described it this way: “I think she can keep up for longer because there is less pain” (parent 7). Furthermore, some parents highlighted that the increased energy motivated their child to initiate activities. One parent exemplified this by saying: “She has started taking the bike out by herself. We have encouraged her for a long time, and now she feels confident on the bike…” (parent 19). In addition to improved activities, many parents also connected the increase in energy to their child’s ability to participate more fully. One parent shared: “Lately, she has had so much energy to join afternoon and overnight activities, and she visits friends and does things she couldn’t do before” (parent 13). Another parent described how it made family life easier, as they could participate in activities more spontaneously without having to plan everything in advance:

*“Before the treatment we somehow couldn’t make appointments because she was so tired in the afternoons. She had to relax….She has so much energy now…That means a lot, because we can take part in activities, we do not have to sit home with her. We also know that she’ll be fine in the kindergarten the day after”.* (parent 3).

##### Theme 2b: Children’s Perceptions

- Category 1: They say

Most of the children depended on their parents’ assessments to determine if the treatment had any noticeable impact. They expressed a hopeful attitude, relying on their parents’ observations and trusting their judgment. One child remarked: “They say that I walk more straight with my foot after the treatment. I hope they are right because I don’t feel it myself” (child 2). Some children did not perceive any difference after the treatment, and one child said: “We do this every year, so I don’t know if not doing it would make any difference” (child 18).

- Category 2: Can do more

While many children did not perceive any effects of the treatment, there were others who noticed a connection between the changes in their muscles and their abilities. One child described it by saying, “My foot has become stronger, and I can walk for longer without getting tired” (child 20). Some also appreciated being able to keep up with other children, as expressed by the same child: “I can keep up with the others, because I don’t get tired as quickly” (child 20).

##### Interpretation of Parent’s and Children’s Perceptions 

Many parents reported improvements in their child following the treatment, which had a positive impact on the entire family. They observed a noticeable increase in the child’s energy levels, enabling them to engage more actively with other children. Additionally, they noticed that the child began initiating new activities. From a phenomenological perspective, these observations can be interpreted as the child’s heightened energy facilitating an outward shift of attention. Their focus turned towards the external world, while the body took a more background role. Merleau-Ponty indicates that as embodied beings, our attention naturally gravitates towards the possibilities presented by our bodies and the invitations and encouragements offered by our surroundings [40]. After the treatment, it can be inferred that some children were more readily able to respond to these “invitations”. For instance, they might “accept an invitation” to go for a bicycle ride due to the new possibilities in the body. Our actions and perceptions are motivated by the interplay between our bodies and the surrounding environment [40]. When we experience limitations in our bodily abilities, such as difficulty in walking or running, it alters our perception of both the environment and ourselves. Therefore, positive changes in the body can contribute to an improved sense of well-being by opening new possibilities for engagement with the world.

In this context, the reported increase in energy and the child’s ability to participate more actively can be viewed as indicators of enhanced well-being resulting from the treatment. Drawing from a phenomenological perspective, one my argue that the positive bodily changes expanded the child’s possibilities for actions and interactions, allowing them to respond to the world in a more fulfilling manner.

### 3.3. Quantitative Results

The measured outcomes showed both positive (dark gray) and negative (light gray) effects from baseline to P1 (diff P1) in both groups. Positive effects are effects above MCID and negative effects are effects below MCID, in the opposite direction (i.e., child 6 had a 42 m decrease in WC from baseline to P1). Most positive effects were observed in group P, especially in EC and COPM performance. Most negative effects were observed in WC regardless of group, which means that they had shorter walking distance at P1 than at baseline, see Table 3. Seven of the children had not received BoNT-A treatment previously, Table 1. This had no significance for the results.

### 3.4. Comparing and Contrasting Qualitative and Quantitative Results 

Both groups were assessed with the same outcome measures. Most positive effects (dark grey) were measured in group P. By contrast, the interview data revealed that the informants (parents and children) in this group did not perceive any effect. On the other hand, most participants in group B perceived a treatment effect. This suggests that the parents and the children were able to perceive whether or not they had received the treatment, and that the perceived effect was somewhat more consistent than the measured effect in this sample. In addition, when parents perceived an effect, they also reported that their expectations of the treatment were fulfilled. When an effect was perceived, expectations were met in 7 out of 8 cases (both groups). Most often, the expectation of treatment and the perception of the effect were related to increased energy, see Table 4.

## 4. Discussion

Measurement and perception may provide complementary insights into the effectiveness of a treatment. In our study, we aimed to investigate the correspondence between expected, perceived, and measured effects of the treatment, BoNT-A, compared to saline (placebo). However, we did not observe any correspondence between these factors, assessed after four weeks. Interestingly, the perceptions of the treatment effects were found to be more consistent with the actual treatment (BoNT-A or saline) than the measured outcomes within our sample. Furthermore, there seemed to be a connection between treatment expectations and the perceived effects, often associated with a reduction in energy expenditure. Notably, there was a distinction between the perspectives of parents and children. Children tended to view their parents as experts in the treatment process and expressed fewer expectations and perceptions regarding the treatment. To our knowledge, our findings provide new insight into the importance of considering subjective experiences and involving children in the treatment process.

### 4.1. Measured and Perceived Treatment Effect

The measured outcomes displayed variability and indicated a mix of positive and negative effects from baseline to P1 in both groups. Several factors may have contributed to this inconsistency, including actual variation in treatment effects, the timing of assessments, and the reliability and validity of the measurement methods utilized. 

Variability in the response to BoNT-A treatment among patients is well documented, both between individuals and between treatments within the same patient. Factors such as age, concomitant therapies used alongside BoNT-A treatment, and the number of previous injections have been suggested as potential contributors to this variability [43]. In particular, the first two injections have been observed to effectively alleviate spasticity [44]. These are factors that may account for some of the variability in treatment effects observed in our sample.

The timing of the assessments may also have contributed to the observed variability. In our study, we conducted the first assessment (P1) four weeks after the treatment. However, this may have been too early to both measure and perceive improvement. Matsuda et al., 2018 [45], in their investigation of gait function over time after BoNT-A treatment, concluded that the maximum improvement in gait function does not occur during the early stage but rather approximately two months after the treatment. Similarly, Giuliani et al. 1991 [46] reported that immediate changes in abnormal motor patterns are not observed when spasticity is reduced with selective dorsal rhizotomy. Therefore, significant changes in motor function may not occur solely by reducing spasticity; some degree of exercise is necessary to enhance gait function. Consequently, some time is required to improve overall function. Additionally, we observed some negative effects four weeks after the treatment, potentially attributed to muscle weakness. The primary objective of BoNT-A treatment is to reduce spasticity, which is primarily achieved by decreasing the active force production of muscles affected by BoNT-A [43]. Another explanation for the negative effects on the calf muscles following the injection could be related to its influence on the proximal joints. For instance, injection into the gastrocnemius muscle has been reported to temporarily exacerbate hip joint motion. The initial excessive hip flexion observed was an attempt to compensate for the improved knee position and obtain better stability [47].

Furthermore, the utilization of reliable and valid outcome measures is crucial when assessing treatment effects. Research conducted by Brehm et al., 2007 [33] and Himuro et al., 2017 [34] emphasized the necessity of evaluation in larger sample sizes among children with CP, specifically in the domains of EC and WC. However, standardized measurements may not capture nuances and complexities that are perceived as important by children and parents. A new promising questionnaire, The Gait Outcomes Assessment List (GOAL), was developed specifically to serve this purpose for ambulatory children with CP. The GOAL questionnaire provides both the means to find out what patients and parents are hoping to achieve from these interventions and whether these goals are met [48,49].

In our sample, we did not measure any significant changes in energy cost during walking after BoNT-A treatment. However, the parents and the children described changes such as being *less stiff and stumbling*, having *more energy*, and having the *ability to do more*. Their perceptions went beyond the narrow focus on walking itself and encompassed the broader functional aspects that walking enables, such as increased activity and participation. This emphasizes the importance of considering individual differences, personal preferences, and unique circumstances when evaluating treatment effects. 

### 4.2. The Impact of Expectations to Perceived Treatment Effects

In our study, we observed that expectations of the treatment had an impact on the perceived effects. Specifically, an anticipated reduction in energy expenditure was associated with a perception of having more energy. This finding aligns with existing literature that highlights the influence of expectations on perceived treatment outcomes [50,51]. A systematic review by Mohammed et al., 2020 [52] supported this notion, reporting that patients with higher expectations of treatment effects tended to experience better outcomes compared to those with lower expectations who showed lesser improvement. This suggests that expectations can significantly affect the perceived effectiveness of a treatment.

Contextual factors, such as healthcare providers’ communication, treatment settings, prior experiences, and social influences, can shape expectations [23,53]. Positive and supportive communication from healthcare providers, for instance, has been shown to enhance positive expectations and subsequently influence perceived treatment effects [53]. In our study, the expectations related to reduced energy expenditure may have been influenced by the participants’ awareness of the main outcome measure, reduced energy cost. Understanding the impact of expectations on perceived treatment effects is crucial for both healthcare providers and researchers. By acknowledging and managing expectations, it is possible to enhance the overall treatment experience and optimize patient outcomes [50,51]. Improved communication and addressing patient expectations can contribute to a positive therapeutic alliance, which may lead to better treatment adherence, satisfaction, and overall treatment outcomes [54].

### 4.3. Diverse Perspectives of Parents and Children 

Many parents in our study expressed a desire to prevent secondary problems in their children with CP. While the primary goal of BoNT-A treatment is to reduce spasticity, its potential for preventing or minimizing secondary problems is still being researched and discussed. A study by Desloovere et al., 2007 [55] suggested that gait kinematics were more normal one year and ten months after the initial BoNT-A injection. This implies that children treated with BoNT-A have a gait pattern that is less affected by bony deformities commonly seen in spastic CP. Based on these results, BoNT-A injections can be considered as a treatment approach aiming to decrease the likelihood of secondary problems at an early age and reduce the need for complex surgeries later on. However, other long-term follow-up studies have reported conflicting results [56,57]. Tedroff et al., 2009 [57] suggested that contractures continue to develop in the long term despite the reductions in muscle tone achieved with BoNT-A. However, in a later study, Tedroff et al., 2010 [56] demonstrated that early treatment with BoNT-A in children with spastic CP may decrease muscle tone and decelerate contracture development after 3.5 years. The uncertainty regarding the preventive effects of BoNT-A treatment should be discussed with parents, since their motivation for treatment often revolves around preventing future difficulties. 

The children, on the other hand, did not focus on preventing secondary problems. They primarily focused on the pros and cons of the treatment, exploring new possibilities, or were content with their bodies as they are. The diverse perspectives of parents and children are also described in other studies [58,59]. Schiariti et al., 2014 [60] conducted interviews with parents and their children regarding their strengths and limitations in functioning. Children talked more frequently about abilities, whereas caregivers talked more about the limitations and broader issues facing their child. Listening to both children and parents is therefore important in clinical practice. In our study, it appeared the children assumed a passive role and regarded their parents as experts. This suggests that the child’s voice may receive insufficient attention in the treatment process, and that child-centered care is not consistently prioritized.

### 4.4. Strengths and Limitations

To the best of our knowledge, this study is the first to incorporate the perspectives of both children and parents in the evaluation of the treatment effect of BoNT-A. It also encompasses objective measurements and subjective perceptions of outcomes, while employing separate analyses prior to ascertaining whether the child received BoNT-A or saline. However, it is worth noting that the evaluation conducted four weeks post-treatment might be considered somewhat premature for detecting significant changes in the measured outcomes.

## 5. Conclusions

Our study aimed to explore the correspondence between expected, measured, and perceived effects of BoNT-A or saline treatment. However, after four weeks, we did not find any correspondence between these factors. Interestingly, perceptions of treatment effects were more consistent than the measured outcomes. We also observed a correspondence between treatment expectations and perceived effects, often related to reduced energy expenditure. Children tended to view their parents as treatment experts and had fewer expectations and perceptions themselves. These findings support the importance of promoting child-centered care, which entails actively listening to children’s expectations and perceptions during the treatment process.

Future research should include patient-reported outcome measures for children with CP that specifically evaluate the efficacy of treatment as judged by the parents and children. Additionally, further understanding of the long-term effects of BoNT-A treatment is necessary.

## Figures and Tables

**Table 1:** Demographic characteristics of the children and their parents.

Id	Age	Gender	GMFCS	UL/BL	Parent	BoNT-A Earlier
1	6	m	I	BL	mo	no
2	9	m	I	UL	fa	yes
3	4	f	I	UL	mo	no
4	4	f	I	UL	fa	no
5	11	f	I	UL	mo	no
6	8	f	I	UL	mo	yes
7	9	f	I	UL	fa	no
8	10	m	I	UL	mo	yes
9	7	f	I	UL	fa	no
10	11	m	I	UL	fa	yes
11	15	m	II	UL	fa	no
12	8	m	I	UL	mo	yes
13	12	f	II	UL	mo	yes
14	10	m	I	UL	mo	yes
15	7	f	I	UL	mo	yes
16	14	m	I	UL	fa	yes
17	8	m	I	UL	mo	yes
18	10	m	I	UL	fa	yes
19	9	f	I	UL	mo	yes
20	10	f	II	UL	mo	yes

Gender; male (m)/female (f), Gross Motor Function Classification System (GMFCS), Unilateral (UL)/bilateral (BL), Parent; mother (mo)/father (fa), BoNT-A earlier in foot.

**Table 2:** Themes and categories.

Theme	Category
1a. Expectations to treatment parents	1. Approaching normality
	2. Saving energy
1b. Expectations to treatment children	1. Just doing it
	2. Pros and cons
2a. Perceived effect parents	1. Less stiff and stumbling
	2. More energy
2b. Perceived effect children	1. They say
	2. Can do more

**Table 3:** Measures at baseline and P1, and change scores (diff 1).

ID	Group	Age	Gender	GMFCS	UL/ BL	EC Baseline	EC Diff 1	WC Baseline	WC Diff 1	COPM per Baseline	COPM per Diff 1	Pain int Baseline	Pain int Diff 1
1	P	6	m	I	BL	5.85	−1.14	97	−2	3.0	1.0	4	0
2	P	9	m	I	UL	3.38	0.96	100	0	5.3	1.0	2	missing
4	P	4	f	I	UL	missing	missing	61	−6	4.7	2.0	4	−2
5	P	11	f	I	UL	3.89	−0.15	115	5	3.7	1.6	1	0
6	P	8	f	I	UL	6.43	−0.53	125	−42	6.3	3.0	1	missing
8	P	10	m	I	UL	5.64	−0.14	111	−17	2.0	2.5	1	3
9	P	7	f	I	UL	5.90	−0.08	95	0	5.3	0.0	3	−2
10	P	11	m	I	UL	4.42	−0.85	93	missing	5.3	0.6	3	−2
17	P	8	m	I	UL	4.67	−0.66	114	−2	5.0	2.0	1	0
18	P	10	m	I	UL	4.76	−0.18	99	−2	7.0	−1.0	1	2
19	P	9	f	I	UL	4.94	1.54	113	0	3.5	1.5	3	0
3	B	4	f	I	UL	missing	missing	89	5	5.0	0.0	2	2
7	B	9	f	I	UL	5.84	0.25	82	−12	2.0	0.7	1	0
11	B	16	m	II	UL	4.56	0.29	84	7	5.0	3.0	2	1
12	B	8	m	I	UL	5.05	missing	105	missing	5.7	missing	5	missing
13	B	12	f	II	UL	4.36	0.14	113	−13	5.5	−0.5	1	0
14	B	10	m	I	UL	4.62	0.72	119	0	6.3	−5.0	1	missing
15	B	7	f	I	UL	6.41	−1.58	77	18	3.0	1.0	5	missing
16	B	14	m	I	UL	3.06	0.10	114	−7	3.5	−1.0	2	1
20	B	10	f	II	UL	5.93	0.08	97	−3	7.0	−1.0	2	0

*P*; Placebo group, *B*; BonT-A group, *Measured positive effect* (*dark gray*); >Minimum important clinical difference (MICD), *Measured negative effect* (light gray); >Minimum important clinical difference (MICD) in the opposite direction, *Energy cost* (*EC*); J/kg/m: Negative value means reduced energy cost, *Walking capacity* (*WC*); Positive value means improvement in meter, *Canadian Occupational Performance Measure*, *performance* (*COPM*, *per*)*:* Positive value means improvement. *Pain intensity* (*PI*); 1–6: Negative value means less pain,

**Table 4:** Quantitative and qualitative data in group P and group B.

ID		Measured Positive Effect	Measured Negative Effect	Perceived Effect Parents	Perceived Effect Children	Expectations Met Parents	Expectations Met Children
Placebo group (P)	1	EC (−1.14)		yes	unsure	energy+	
	2		EC (0.96)	yes	yes	normal *	energy+
	4	COPM (2), Pain int (−2)	WC (−6)	no	too young		
	5			no	no		
	6	EC (−0.53), COPM (3)	WC (−42)	no	no		
	8	COPM (2.5)	WC (−17), Pain int (3)	no	no		
	9	Pain int (−2)		no	unsure		
	10	EC (−0.85). Pain int (−2)		no	no		
	17	EC (−0.66), COPM (2)		no	no		
	18		Pain int (2)	no	no		
	19		EC (1.54)	yes	no		
BoNT-A group (B)	3		Pain int (2)	yes	too young	energy+	
	7		WC (−12)	yes	unsure	energy+	
	11	WC/(7), COPM (3)		no P1	no P1		
	12 *			no P1	no P1		
	13		WC (−13), COPM (−5)	yes	no	energy+	
	14		EC (0.72)	no	unsure		
	15	EC (−1.58), WC (18)		yes	no P1	pain-	
	16		WC (−7)	no	no		
	20			yes	yes	stifness-	

*Measured positive effect* (*dark gray*); >Minimum important clinical difference (MICD), *Measured negative effect* (*light gray*); >Minimum important clinical difference (MICD) in the opposite direction, *Energy cost* (*EC*); J/kg/m: Negative value means reduced energy cost, *Walking capacity* (*WC*); Positive value means improvement in meter, *Canadian Occupational Performance Measure*, *performance* (*COPM*, *per*): Positive value means improvement. *Pain intensity* (*PI*); 1–6: Negative value means less pain, energy+; more energy, normal *; more normal (normal development, be like the other), pain-; Less pain, stiffness-; Less stiff in the muscles, * Dropout P1. The number in the brackets is the difference from baseline.

## Data Availability

The data presented in this study are available upon request from the corresponding author. The data are not publicly available due to ethical reasons, public sharing of data was not specifically consented by participants.
